# Exosomes: mediators regulating the phenotypic transition of vascular smooth muscle cells in atherosclerosis

**DOI:** 10.1186/s12964-022-00949-6

**Published:** 2022-10-11

**Authors:** Jiali Yao, Linqian Cai, Yingrui Chen, Jie Zhang, Wenwen Zhuang, Jingyan Liang, Hongliang Li

**Affiliations:** 1 Institute of Translational Medicine, Medical College, Yangzhou University, Yangzhou, 225009 Jiangsu China; 2 Department of Neurology, Afliated Hospital of Yangzhou University, Yangzhou, 225001 China; 3 Jiangsu Key Laboratory of Experimental and Translational Non-Coding RNA Research, Yangzhou University, Yangzhou, 225009 Jiangsu China

**Keywords:** Atherosclerosis, Vascular smooth muscle cells, Phenotypic transition, Exosomes

## Abstract

Cardiovascular disease is one of the leading causes of human mortality worldwide, mainly due to atherosclerosis (AS), and the phenotypic transition of vascular smooth muscle cells (VSMCs) is a key event in the development of AS. Exosomes contain a variety of specific nucleic acids and proteins that mediate intercellular communication. The role of exosomes in AS has attracted attention. This review uses the VSMC phenotypic transition in AS as the entry point, introduces the effect of exosomes on AS from different perspectives, and discusses the status quo, deficiencies, and potential future directions in this field to provide new ideas for clinical research and treatment of AS.

**Video Abstract**

**Supplementary Information:**

The online version contains supplementary material available at 10.1186/s12964-022-00949-6.

## Introduction

Atherosclerosis (AS) is a chronic inflammatory disease with complex pathogenesis that involves a variety of cells, such as vascular smooth muscle cells (VSMCs), endothelial cells (ECs), and macrophages. In the classical view of the mechanisms of AS, VSMCs are believed to promote AS via the transformation of contractive VSMCs to synthetic VSMCs, migration from the middle membrane to the intima, and the proliferation and deposition of matrix proteins, which increase plaque thickness. Increasing evidence indicates that the phenotypic transition of VSMCs is a core event in the pathophysiology of many cardiovascular diseases (including AS and restenosis after angioplasty) [1]. Therapeutic strategies against the VSMC phenotypic transition may improve pathological conditions related to VSMCs and provide a new therapeutic target for preventing and treating AS.

The exchange of information between cells is essential for maintaining cell function. Experimental and clinical studies have reported that extracellular vesicles released by cells help complete inter-cell communication through direct contact, internalization, fusion with the plasma membrane, and endocytosis [2]. As one of the subgroups of extracellular vesicles, exosomes promote the transfer and exchange of microRNAs, mRNAs, and lncRNAs between cells and tissues, playing a critical role in the migration and differentiation of cells. Exosomes also play a crucial role in the pathophysiological processes of many diseases [3] and can be used as a diagnostic biomarker as well as a therapeutic target for AS.

In this review, we introduce the role of exosomes in the phenotypic transition of VSMCs and the effect of related pathways on AS from different perspectives to provide a new direction for preventing and treating AS.

## Biological properties of exosomes

Extracellular vesicles are lipid-bound vesicles secreted by cells into the extracellular space and consist of three subgroups: exosomes, microvesicles, and apoptotic vesicles [4, 5].

Exosomes were first discovered in reticulocytes in 1983[6]. The classical markers of exosomes are CD9, CD63, and CD81 [7]. Exosomes affect recipient cells more efficiently than that by intercellular contact or by secreted factors, and they originate from intranuclear bodies. Specifically, exosomes are produced when intracellular lysosomal particles invaginate to form polyvesicular bodies that fuse with the cytoplasmic membrane and are released into the extracellular compartment [7]. Although the biological origins and clinical application pathways of microvesicles and exosomes are similar, exosomes have received more attention. Exosomes are more widely present in various body fluids, such as blood and urine, which facilitates noninvasive fluid biopsies in patients to diagnose disease and monitor the patient’s response [8]. Previous studies have shown that exosomes can be used as carriers to transport drugs into target cells, similar to nanomaterials [9, 10]. Moreover, exosomes have a longer circulating half-life and are better tolerated by humans than other drug delivery systems [10]. The biostability and clearance patterns of exosomes are similar to those of synthetic nanoparticles, and some studies have shown that exosomes can evade partial attack by the immune system and remain in circulation for a long time. The immunogenicity of autologous or allogeneic exosomes is negligible, as repeated injections of autologous exosomes do not provoke a significant immune response in mice [11], and most exosomal agents used clinically are derived from allogeneic cells [12]. These findings may be due to the CD47-mediated effect of protecting exosomes from phagocytosis by monocytes and macrophages [13]. Apoptotic vesicles are released by dead cells. Their composition and proteomic profile are significantly different to those of exosomes, and their biological role is far less extensive [14].

It is currently believed that there are three main mechanisms to achieve exosomes mediated signaling [15]. (a) The first and most direct type of intercellular communication is the ligand-receptor interaction. Juxtacrine signaling will be delivered between exosomes and receptor cells through direct contact. This discovery has functioned as a revelation in treating particular diseases. For example, pretreating ovarian cell-derived exosomes with proteinase K or trypsin to degrade exosomal transmembrane proteins may eliminate their uptake by cancer cells [16, 17]. No single specific way has been shown for this ligand-receptor interaction. After the direct contact between exosomes and receptor cells, exosomes can trigger intrinsic signaling in the recepor cells [18], or the exosomes pass through the receptor cells and target other cell types [22]. (b) The second type of mechanism is that of indirect communication. Soluble ligands are cleaved from exosomal transmembrane proteins, which interact with receptors on the surface of target cells and activate a variety of signaling pathways. Complement molecules or miRNAs shed from cells by exosomes act as interventional targets or introduce substances that act as protectors against disease [23]. (c) The third type is that of endocytosis, which includes lattice-protein-mediated and/or niche protein-dependent endocytosis, macropinocytosis, phagocytosis, and lipid raft-mediated endocytosis [15, 24]. Signals delivered by exosomes are internalized by receptor cells through an endosomal mechanism [19–21]. Deformation of the cell membrane induced by latticin leads to the formation of an inwardly-facing bud and the development of a larger vesicle that matures and then contracts, thereby delivering the exosomal contents to the recipient cells [24]. The lattice proteins take up exosomes by invaginating the plasma membrane and are subsequently internalized by recipient cells after kinesin is activated. The macrocytic drinking action is characterized by the formation of a ruffled extension of the plasma membrane around the extracellular space, which includes the extracellular fluid and the components that will be internalized by the cell. Phagocytosis is dependent on the association between plasma membrane receptors and vesicle ligands. Lipid raft-mediated endocytosis is related to the structure of lipid rafts. Lipid rafts are formed from microstructural domains rich in cholesterol and sphingolipids and rich in protein receptors [25]. Although the interactions between exosomes and recipient cells may not be directly interfered with, some properties of exosomes have been used to introduce new ideas for therapy. Because of their lipid nature and the presence of specific ligands on their surface, exosomes are expected to be used as carriers for the therapeutic delivery of RNAs, peptides, and synthetic drugs [26].

Exosomes contain miRNA, mRNA, IncRNA, proteins, and lipids. Among them, miRNAs have attracted more attention than other contents by virtue of their function in regulating gene expression. Exosomes significantly regulate cell growth and metabolism through transcriptional repression of gene expression. When absorbed by specific cells, exosomes may play a role locally or at a distance, providing autocrine or paracrine signals, or inhibiting mRNA translation by transferring miRNA to target cells, which may lead to the production of new proteins, resulting in a protective or damaging response [6, 27, 28]. For example, bone-derived exosomal miRNAs are thought to be important in regulating the expression of genes involved in differentiation and communication between multiple cell types [29]. Exosomal miRNAs derived from immune cells are thought to be involved in cardiovascular disease. Exosomal miRNAs from cardiomyocytes or stem cells play a role in cardiac repair and regeneration [30, 31]. Exosomes from fibroblasts promote myocardial hypertrophy through miRNA acting on target factors [32]. It is evident that exosomal miRNAs are important and contribute to the exchange of information between cells. Therefore, this review is mainly focused on the role of miRNAs in exosomes.

The role of exosomes in intercellular communication is expected to alter the transcriptome of recipient cells and have key effects on the process of VSMC-mediated AS. As shown in Table 1, we summarized the information related to the exosomal components involved in this review.

**Table1:** Characteristics of different exosomal components in cardiovascular diseases

Components	Sources	Target cells	Effects	Signaling pathways	Associated pathologies	Refs
miR-92a-3p	HUVECs (Human umbilical vein endothelial cells);ECs	ECs; HUVECs;	ECs proliferation, migration and angiogenesis↑; ECs oxidative stress, inflammation and dysfunction↑; HUVECs apoptosis↑proliferation↓;	SIRT6/MAPK; MAPK/NF-κB	Atherosclerosis; Catheter-related thrombosis	[45, 97, 98]
miR-21-3p	ECs; Macrophages	VSMCs	VSMCs migration and proliferation↑; VSMCs phenotypic conversion↑; ECs pro-inflammatory cytokine levels↑;	AGE/RAGE	Atherosclerosis; Diabetic atherosclerosis	[47, 99]
miR-663	VSMCs; PASMCs (Pulmonary artery smooth muscle cells); ECs	VSMCs; PASMCs	VSMCs differentiation↑, proliferation and migration↓; PASMCs proliferation, migration and collagen synthesis↓;	JunB/Myl9; TGF-β1/SMAD2/3	Atherosclerosis; Pulmonary arterial hypertension	[50, 100, 101]
miR-222	M1 macrophages; ECs;	VSMCs; Cardiomyocytes	VSMCs proliferation and migration↑; Cardiac fibrosis↓; Cardiomyocytes proliferation↓ and apoptosis↑	Wnt/β-catenin; PI3K/AKT	Atherosclerosis; Dilated cardiomyopathy; Myocardial ischemia/reperfusion injury	[52, 102, 103]
miR-155-5p	VSMCs Adventitial fibroblasts	VSMCs; HUVECs;	VSMCs proliferation, migration, calcification and invasion↓; HUVECs proliferation, migration and invasion↓;	TGF-β1/Smad2/3	Atherosclerosis; Vascular calcification	[53, 104, 105]
LncRNA H19	HUVECs; VSMCs;	VSMCs	VSMCs osteoblast phenotypic transition↑; HUVECs proliferation↑ apoptosis↓; VSMCs proliferation↓apoptosis↑;	MAPK/NF-κB; WNT/β-catenin	Atherosclerosis; Arterial calcification	[57, 106, 107]
miR-143/145	VSMCs; ESCs (Embryonic stem cells);	VSMCs	VSMCs differentiated phenotype↑;	Jag-1/Notch	Atherosclerosis	[49, 74, 108, 109]
miR-103-3p	VSMCs; Cardiomyocytes	VSMCs; Cardiomyocytes	VSMCs osteoblast phenotypic transition↑; Cardiomyocytes apoptosis↓proliferation↑;	GSK3β/β-catenin;	Vascular calcification; Acute myocardial infarction	[57, 110]
miR-133a	VSMCs;	VSMCs; Cardiomyocytes	VSMCs Osteogenic Differentiation↓; Myocardial collagen deposition↓, Myocardial fibrosis↓;	TGF-β1	Atherosclerosis; Myocardial infarction	[60, 111]
miR-204/miR-211	VSMCs	VSMCs; Cardiomyocytes	Vascular calcification and ageing↓; Cardiac tissues apoptosis level↓;	SIRT1/p53	Atherosclerosis; Myocardial infarction;	[61, 112]
miR-34a	ECs; MSCs;	VSMCs; HAECs (Human aortic endothelial cells); MSCs; Cardiomyocytes	ECs growth↑apoptosis↓; MSCs autophagy and apoptosis↑; cell activity↓;	SIRT1/FoxO3a; Wnt/β-catenin	Atherosclerosis; Myocardial infarction; Type 2 diabetes mellitus	[62, 113–115]
miR-106a	VSMCs	VSMCs;	VSMCs apoptosis↑	Foxo1/Vcam1	Abdominal aortic aneurysm	[66, 147]
miR-106a-3p	Macrophages	VSMCs;	VSMCs proliferation↑apoptosis↓;	CASP9/Caspase	Atherosclerosis	[67]
miR-106a-5p	HUVECs; ECs	HUVECs; VSMCs	HUVECs viability↓apoptosis↑; VSMCs proliferation↑;	Foxo1/Vcam1	Atherosclerosis; Type 2 diabetes mellitus	[68, 148]
circHIPK3	Cardiac fibroblasts; Cardiomyocytes	VSMCs; Cardiac fibroblasts; Cardiomyocytes; CMVECs (Cardiac microvascular endothelial cells)	Cardiac fibroblasts proliferation, migration and phenotypic transition↑; Cardiomyocytes proliferation↓apoptosis↑;	PI3K/AKT; miR-29a/IGF-1; miR-106a-5p/Foxo1/Vcam1	Ischemic heart disease; Myocardial infarction; Ischemia–reperfusion injury	[68, 144–147]
miR-125b-5p	VSMCs	VSMCs; Cardiomyocytes; Pancreatic β-cells	Cardiomyocytes apoptosis↓; Pancreatic β-cells proliferation↓apoptosis↑;	JNK	Acute myocardial infarction; Type 2 diabetes mellitus	[70, 142, 143]
circRNA0077930	HUVECs	VSMCs	VSMCs senescence↑;	/	Diabetes mellitus; Oronary heart disease	[77]
miR-622	HUVECs	VSMCs; Cardiomyocytes	Cardiomyocytes apoptosis and inflammatory reaction↑;	Kras CeRNA	Acute myocardial infarction;	[77, 141]
miR-155	VSMCs; Cardiomyocytes	VSMCs; ECs; THP-1 macrophages;	VSMCs migration and over proliferation↓; Cardiomyocytes apoptosis and cardiac fibrosis↑;	ERK1/2; Nrf2/HO-1	Atherosclerosis; Diabetic cardiomyopathy	[79, 138–140]
miR-147	MSCs	VSMCs; H9c2 cells; Macrophages	Myocardial inflammation and apoptosis↓; inflammatory response↓;	NF-κB; TGF-β	Abdominal aortic aneurysm; Myocardial infarction;	[87, 136, 137]
miR-125b	MSCs; BMSCs (Bone marrow mesenchymal stem cells);	VSMCs; Ischemia reperfusion myocardium cells; Cardiomyocytes	VSMCs proliferation and migration, neointima formation↓; Ischemia reperfusion myocardium cells viability↑ inflammation and apoptosis↓; Cardiomyocytes apoptosis↓;	P38/Sirtl/P53; Nrf2/HIF-1a	Atherosclerosis; Myocardial ischemia reperfusion injury; Acute myocardial infarction	[55, 118, 119, 135]
miR-29b	HAVICs (Human aortic valve interstitial cells)	VSMCs; Osteoblasts; HUVECs; Cardiomyocytes	HAVICs osteoblastic differentiation and calcification↑; ECs inflammation↓; Myocardial fibrosis and cardiac hypertrophy↓;	SPRY1/MAPK; Notch; Wnt3/β-catenin/Smad3	Atherosclerosis; Myocardial Infarction; Calcific aortic valve diseases	[58, 132–134]
miR-128-3p	Atrial fibroblasts; VSMCs	Atrial fibroblasts; VSMCs	Atrial fibroblasts proliferation, collagen production↑; VSMCs proliferation and migration↓;	TGF-β1/Smad; FOXO4/MMP9;	Atrial fibrillation; Atherosclerosis	[75, 120, 121]
miR-223-3p	Cardiomyocytes;	VSMCs; Cardiomyocytes; CMECs (cardiac microvascular endothelial cells)	CMECs apoptosis↓; CMECs migration, proliferation and angiogenesis↓	PI3K/Akt; RPS6KB1/hif-1a	Ischemic heart diseases; Acute myocardial infarction	[59, 128–130]
LncRNA LIPCAR	THP-1 cells; Atrial fibroblasts	VSMCs; HUVECs; Atrial fibroblasts	HUVECs proliferation, angiogenesis, apoptosis and oxidative stress↓; Atrial fibroblasts proliferation↑;	TGF-β/Smad;	Atherosclerosis; Atrial fibrillation	[51, 131]
miR-221-3p	HUVECs	VSMCs; HUVECs; Cardiomyocytes	HUVECs apoptosis, inflammation, and oxidative stress↓; Cardiomyocyte apoptosis, myocardial injury↓, Inflammatory reaction and oxidative stress↓;	TLR4/NF-κB; NLRP3/ASC/pro-caspase-1	Atherosclerosis; Coronary heart disease	[54, 116, 117]
LINC01005	HUVECs	VSMCs	VSMCs proliferation and migration↑;	/	Atherosclerosis	[75]
miR-223-5p	Plasma	VSMCs	VSMCs viability↓apoptosis↑;	miR-6515-5p/VCAM1	Thromboangiitis obliterans;	[69]
Notch3	HUVECs	VSMCs	VSMCs calcification and aging↑;	mTOR	Diabetes mellitus; Myocardial infarction	[78, 158]
miR-150	EPCs (Endothelial progenitor cells)	VSMCs; EPCs; ECs;	EPCs differentiation and thrombus resolution↑; ECs proliferation, migration↑ vascular remodeling↓	Akt/FOXO1; NF-κB	Type 2 diabetes; Deep venous thrombosis; Acute coronary syndrome	[88, 122–124]
miR-16-5p	Plasma	Cardiomyoblasts; VSMCs	Cardiac cells ER stress and oxidative stress↑	ATF6	Intracranial atherosclerotic disease; Dilated cardiomyopathy	[94, 125]
miR-486-5p	BMSCs	VSMCs; Cardiomyocytes	Cardiomyocytes apoptosis and growth↓apoptosis↑;	PTEN/PI3K/AKT	Myocardial ischemia–reperfusion injury; Cyanotic congenital heart disease	[94, 126, 127]
miR-30c-5p	Ischemia reperfusion myocardial cells;	VSMCs; Macrophages; Ischemia reperfusion myocardial cells; HUVECs	Inflammation and apoptosis↑; Macrophages apoptosis and inflammation↑; HUVECs inflammatory response and apoptosis↓;	NF-κB; Wnt7b/β-catenin	Myocardial ischemia reperfusion injury; Atherosclerosis;	[94, 149–151]
miR-30e	Cardiomyocytes	VSMCs; Cardiomyocytes	Cardiac fibrosis↓; Myocardial ischemia–reperfusion injury↓; Ventricular remodeling↑;	Snai1/TGF-β	Cardiomyopathy; Atherosclerosis; Myocardial ischemia–reperfusion injury	[95, 152, 153]
miR-92a	ECs	VSMCs; Cardiomyocytes	VSMCs proliferation and migration↑; Cardiomyocytes apoptosis↓;	NF-κB; ROCK/MLCK	Diabetes mellitus; Atherosclerosis Myocardial ischemia reperfusion injury	[95, 154–156]
miR-133	Cardiomyocytes	Cardiomyocytes; VSMCs	Cardiomyocytes apoptosis↓; VSMCs proliferation and migration↓; Phenotypic switch and vascular remodeling↓;	MAPK/ERK;	Myocardial infarction; Atherosclerosis	[48, 157]

## Role of the VSMC phenotypic transition in AS

Although different cell types are involved in AS, VSMCs make up the thickest layer of the arterial wall, and their status largely reflects the state of the blood vessels. VSMCs are highly plastic and capable of phenotypic transition in response to different regulatory signals [33]. In normal blood vessels, VSMCs are a highly static and contractile phenotype associated with elevated levels of α-smooth muscle actin (α-SMA), SM 22α, smooth muscle myosin heavy chain, and other contractile marker proteins [34].

After vascular injury, VSMCs lose their contractile phenotype and switch to a synthetic phenotype. Cells of the contractile phenotype are characterized by high expression of contractile genes and low proliferation and migration rates, whereas VSMCs of the synthetic phenotype express lower levels of contractile genes and have higher proliferation and migration rates [35].

In the classical view of the AS mechanisms, the VSMC phenotypic transition is believed to lead to the deterioration of AS. Contractive VSMCs shift to syngenesis, migrate from the middle membrane to the intima, proliferate, and deposit matrix proteins, which have some compensatory significance during the early stage, but can cause a damage reaction and lead to plaque thickening during the late stage. At the same time, synthetic VSMCs express a variety of fatty acid and cholesterol uptake receptors and perform the function of capturing fatty acids and cholesterol and filling the cytoplasm with lipid droplets, thus facilitating the absorption of lipid and the formation of foam cells. Foam cells are swollen vacuolated macrophages filled with lipid inclusions that often accumulate along arterial walls and are characteristic of some disturbed lipid metabolism conditions; foam cells are present during all stages of the development of AS. Studies have shown that 70% of foam cells are derived from VSMCs [36, 37]. In addition, VSMCs can be transformed into a pro-inflammatory and dysfunctional macrophage phenotype, which assumes the function of macrophages, leading to the deterioration of AS [38, 39].

Vascular calcification is a risk factor for the onset of, and death from, AS. The process of vascular calcification is similar to that of osteogenesis and is caused by the transformation of VSMCs into the osteoblast-like phenotype. Osteoblast-like VSMCs secrete bone-related protein biomarkers, which are involved in osteoblast differentiation, maturation, and other osteogenic processes, ultimately leading to calcification of the intima and plaque formation [40–42]. However, the osteoblast-like phenotype transformation of VSMCs can increase plaque stability during the late stage of AS, and prevent plaque rupture, which has positive implications.

In conclusion, the phenotypic transition of VSMCs plays a damaging and protective role during AS. Identifying the regulatory targets between them, to balance or reverse such processes, is a difficult problem.

## Roles of exosomes in the regulation of VSMC proliferation and migration

Exosomes from circulation participate in the pathogenesis of vascular diseases. In one study, plasma-derived exosomes from healthy subjects did not promote the proliferation and migration of VSMCs, while plasma-derived exosomes from peripheral artery disease (PAD) patients promoted the proliferation and migration of VSMCs and inhibited the migration of ECs [43]. This may be related to the finding that they contain miRNAs with different characteristics.

TET2 is a key regulator of the VSMC phenotypic transition. Bo Li et al. showed that low expression of TET2 in exosomes derived from ECs promotes proliferation and migration of VSMCs and intimal hyperplasia after arterial injury [44]. Additionally, other studies have shown that exosomes released by ECs stimulated by oxidative stress enhance proliferation and migration of ECs and promote angiogenesis by reducing the expression of miR-92a-3p in ECs [45]. Interestingly, EC-derived exosomes transfer miR-92A-3p to VSMCs and promote proliferation and migration, exacerbating the inflammatory reaction [46]. In other words, early knockdown of exosomal miR-92a-3p expression promotes the proliferation and migration of ECs for compensatory repair, while it promotes the proliferation and migration of VSMCs, leading to an injury reaction after delivering miR-92a-3p to the VSMCs.

Exosomal miR-21-3p targets the downregulation of PTEN and triggers the NF-kappaB pathway to promote proliferation and migration of VSMCs and accelerate the development of plaque in AS; miR-133 suppresses proliferation and migration of VSMCs by downregulating SP-1, while miR-143/145 suppresses proliferation and differentiation of VSMCs by inhibiting KLF4/5. miR-663 promotes differentiation of VSMCs and inhibits proliferation and migration by reducing the expression of JunB and matrix metalloproteinase (MMP)-9 [47–50]. Exosomes derived from foam cells mediate proliferation and migration of VSMCs through IncRNA LIPCAR, leading to the deterioration of AS [51]. Moreover, exosomes promote proliferation and migration of VSMCs through miR-222; thus, aggravating intimal hyperplasia and vascular restenosis [52].

Fibroblasts are a major producer of the extracellular matrix and are involved in AS. Xing et al. showed that exosomes from outer membrane fibroblasts downregulate the expression of angiotensin-converting enzyme through miR-155-5P, inhibit proliferation and migration of VSMCs, and improve the state of vascular remodeling [53]. Exosomal miR-221-3p derived from adipose tissue is absorbed by VSMCs, which significantly enhances the proliferation and migration of VSMCs by targeting peroxisome proliferator-activated receptor γ coactivator 1a and triggering early vascular remodeling in the context of obesity-related inflammation [54]. The exosomes derived from mesenchymal stem cells transfer miR-125b to VSMCs by inhibiting Myo1e, suppressing the proliferation and migration of VSMCs in vitro, and restraining the proliferation of new intima in vivo [55].

Thus, the different exosomal components regulate the proliferation and migration of VSMCs to different degrees and participate in various processes in AS. Studies on this aspect have the potential to regulate the proliferation and migration of VSMCs and thus capture the progression of AS.

## Roles of exosomes in the regulation of the osteoblast-like phenotypic transition

Vascular calcification is widespread in AS, and the cause of death in most AS patients is plaque rupture, which is mainly related to its components. Studies have shown [56] that the plaque most prone to rupture is composed of a mixture of calcified and uncalcified tissues; that is, early-stage calcification. This plaque is highly unstable but is not prone to rupture after complete calcification. The transformation of VSMCs into osteoblast-like cells is one of the ways that vascular calcification develops. Elucidating the mechanism of phenotypic transdifferentiation of VSMC osteoblasts is the key to diagnosing and treating vascular calcification.

Zhou et al. [57] reported that exosomal LncRNA H19 is highly expressed in calcified cell models, where it promotes the transformation of VSMCs into the osteoblast phenotype by inhibiting miR-103-3p to upregulate the expression of osteoblast-specific markers, such as bone morphogenetic protein-2 and osteopontin. Researchers have found that microRNA profiles in the exosomes derived from calcified VSMCs are significantly altered, using deep sequencing and bioinformatics. For example, miR-125b inhibits VSMC calcification by inhibiting ETS-1, and overexpression of miR-29b accelerates VSMC calcification. miR-128-3p promotes cardiovascular calcification through the Wnt pathway [58]. However, these studies were conducted in a calcification model, and whether this pathway is also applicable to AS needs to be verified by a model closer to AS. Han et al. [59] showed that exosomes convey miR-223-3p to VSMCs to inhibit their osteogenic conversion and vascular calcification in AS by blocking the signaling pathway mediated by interleukin (IL)-6/STAT3; overexpression of exosomal miR-133a inhibits phenotypic transition of VSMCs into osteoblasts, and the application of miR-133a inhibitors promotes this process [60]. Exosomal miR-204/miR-211 inhibits the phenotypic transition from VSMCs to osteoblasts in a paracrine manner [61], while vascular senescence induced by miR-34a promotes the transformation of VSMCs into osteoblasts under high phosphorus conditions [62].

Vascular endothelial growth factor (VEGF) is an important regulator of VSMCs and an indicator of dedifferentiation from VSMCs to osteoblast cells. Progress has been made on the regulation of VEGF exosomal miRNAs [63]. Alkaline phosphatase is another important marker of osteogenesis, according to one study [64]. Exosomes of vascular ECs stimulated by hyperglycemia regulate calcification of VSMCs by upregulating the expression of alkaline phosphatase.

**Fig. 1:**
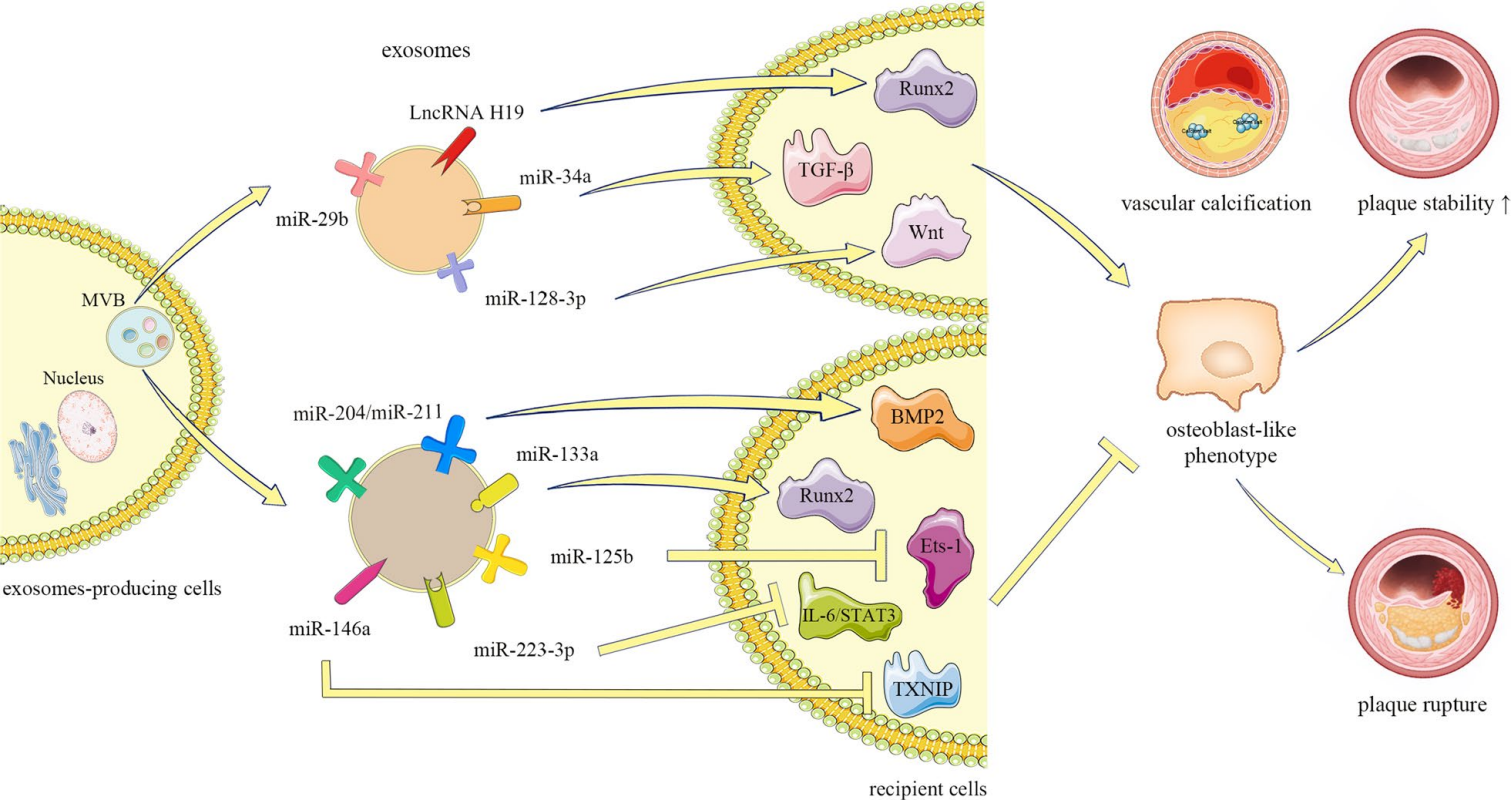
The miR-29b, LncH19/Runx2, miR-34a/TGF-β, and miR-128-3p/Wnt pathways promote the osteoblast phenotype to increase plaque stability. The miR-133a/Runx2, miR-204/miRr-211/BMP2, miR-125b/Ets-1, miR-223-3p/IL-6/STAT3, and miR-146a/TXNIP pathways reduce vascular calcification and senescence by inhibiting the phenotypic transition from VSMCs to osteoblasts and inducing plaque rupture

Thus, exosomal LncRNA H19, miR-103-3p, and miR-133a play a key regulatory role in the phenotypic transition from VSMCs to osteoblasts. Applying specific inducers or blockers to intervene at different stages of AS may help reduce vascular calcification due to aging or plaque rupture events caused by incomplete calcification. However, the problem that still needs to be solved is that no specific indicators are available for the degree of plaque development during the different times in which an intervention would achieve a therapeutic effect (Fig. 1).

## Roles of exosomes in apoptosis of VSMCs in AS

It has been confirmed that apoptosis of VSMCs plays a key role in the development of AS. Excessive apoptosis of late VSMCs damages the structural integrity of plaque, increases instability of the plaque, and leads to plaque rupture, thereby promoting the deterioration of AS. In addition, the phagocytic function of macrophages is inhibited in the plaque environment, leading to the secondary release of inflammatory factors after apoptosis of VSMCs and exacerbation of the inflammatory response [65]. Therefore, it is of great significance to study apoptosis in VSMCs during AS.

The expression of miR-106a in exosomes released by abdominal aortic aneurysm tissues is higher than that in normal tissues, and the enhanced expression of miR-106a promotes apoptosis of VSMCs compared with a control group [66]. However, other studies have reported that THP-1 induced by oxidized low-density lipoprotein (ox-LDL) can be transfected into VSMCs through exosomes to mediate the high expression of miR-106a-3p in VSMCs and directly binds to CASP9 to inhibit the caspase pathway in VSMCs, which alleviates VSMC apoptosis [67]. Moreover, circHIPK3 has been confirmed to be enriched on exosomes derived from mouse aortic smooth muscle and has binding sites for miR-106a-5p, which reduce the proliferation of VSMCs and promote apoptosis through the circHIPK3/miR-106a-5p/Foxo1 axis [68]; exosomal miR-223-5p derived from plasma inhibits VSMC activity and promotes apoptosis by downregulating VCAM1 and IGF1R [69].

Previous studies have shown increased expression of exosomal miR-125b-5p isolated from bone marrow mesenchymal stem cells in mice with AS. miR-125b-5p reduces the inflammatory response, lowers the lipid level, and slows down plaque formation in AS mice by downregulating Map4k4. That study also found that the presence of miR-125b-5p enhances the expression of a-SMA, suggesting that the apoptosis rate of VSMCs in AS mice decreases after the intervention [70].

**Fig. 2:**
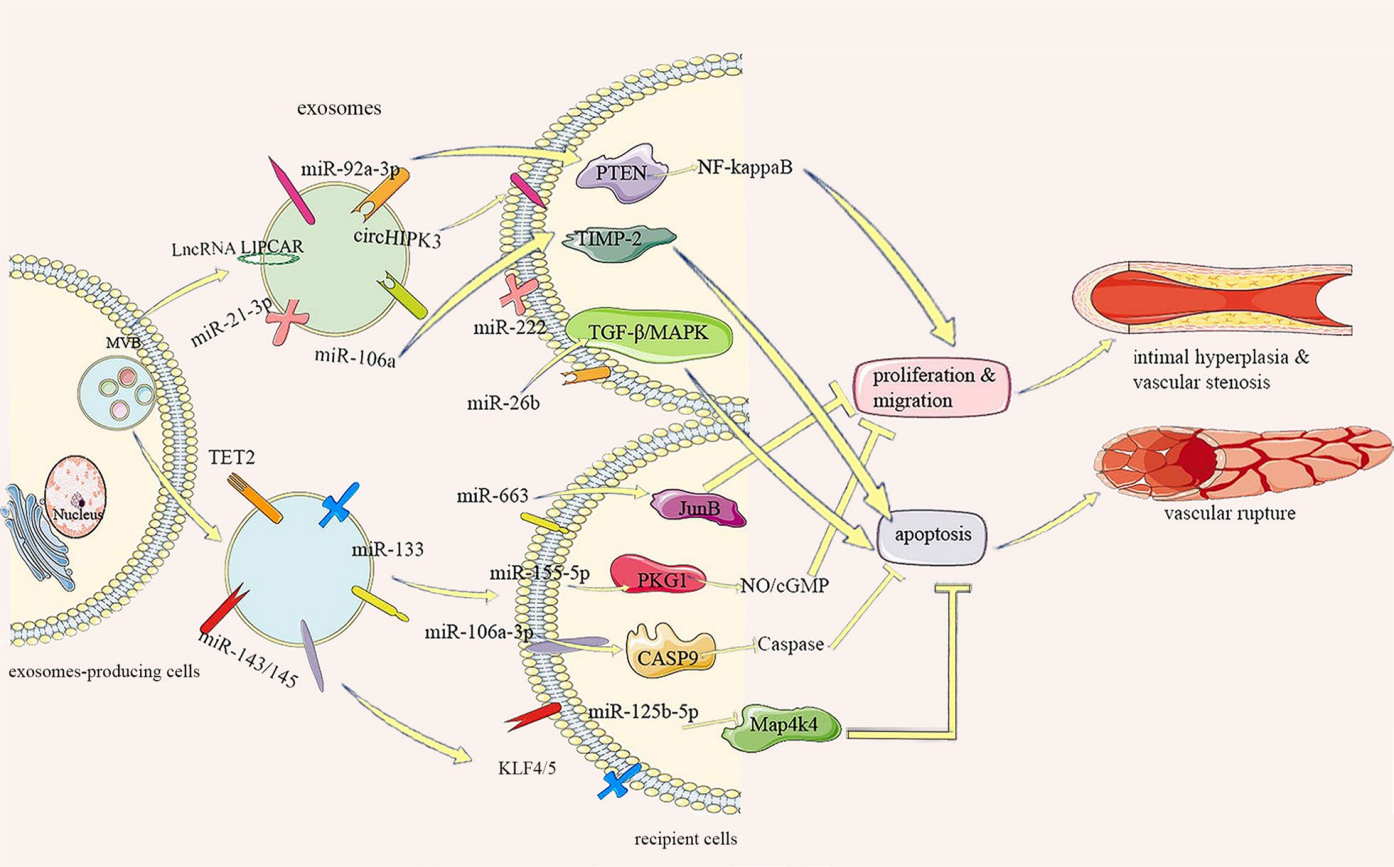
Multivesicular bodies are fused with the cytomembrane and exosomes are released. Exosomes promote the proliferation and migration of VSMCs through the miR-92a-3p/PTEN, miR-21-3p/PTEN/NF-kappaB, miRr-222, and lncRNA LIPCAR pathways, leading to intima hyperplasia. However, the TET2, miR-133, miR-143/145/KLF4/5, miR-663/JunB, and miR-155-5p/PKG1/NO/cGMP pathways improve the pathological process and vascular remodeling by inhibiting proliferation and migration of VSMCs. miR-106A/TIMP-2, circHIPK3/miR-106a-5p/Foxo1, and miR-26b/TGF-β/MAPK pathways promote apoptosis of VSMCs to promote a vascular inflammatory reaction and vascular rupture. miR-106a-3p binds CASP9 to inhibit the caspase pathway in VSMCs. miR-125b-5p downregulates Map4k4, and both inhibit VSMC apoptosis to reduce vascular stenosis and inflammation

All of these studies suggest that regulating the secretion of exosomes to control VSMC apoptosis may be a new target for treating AS. Notably, apoptosis of VSMCs can have different effects at different stages of AS. Previous studies have shown that miRNAs, such as miR-106a, miR-106a-3p, miR-106a-5p, and miR-125b-5p, are mediated by different exosomes from different cell sources, and have important effects on the balance of proliferation and apoptosis of VSMCs. Apoptosis of VSMCs can be regulated and vascular lumen stenosis can be reduced by promoting the secretion of specific exosomes during the early stage. When AS progresses to an advanced stage, reducing secretion with exosomal component inhibitors or antibodies relieves the inflammatory reaction caused by excessive VSMC apoptosis, improves plaque stability, and reduces the incidence of serious cardiovascular events (Fig. 2).

## Roles of exosomes in regulating the interaction between ECs and VSMCs

AS is the result of pathological changes in many types of cells, including ECs, VSMCs, and macrophages. Vascular wall lesions in AS are closely related to ECs and VSMCs. ECs normally regulate vasodilation, proliferation, migration of VSMCs, and intercellular adhesion through molecules that maintain vascular homeostasis [71]. During EC injury, NOD1 upregulates the expression of vascular cell adhesion molecule-1 (VCAM-1) through the RIP2-NF-kappaB axis to promote the recruitment of early leukocytes [72]. In contrast, the endothelium-mesenchymal transformation process induced by transforming growth factor (TGF)-β generates proinflammatory cells, secondary necrotic apoptosis of VSMCs, and unstable plaque growth, which increases the incidence of adverse cardiovascular events [73]. Endothelial dysfunction and excessive proliferation of VSMCs accelerate the deterioration of AS. In previous studies, the independent mechanisms of ECs and VSMCs in AS have gradually been understood, but the regulatory mechanism of AS through the exchange of information between ECs and VSMCs needs further work.

Exosomes act as mediators during cell-to-cell communication. Endothelium-derived exosomes regulated various activities and functions of VSMCs. Studies have shown that the shear response regulator called Kruppel-like factor 2 in ECs significantly upregulates miR-143/145, and these miRNAs are enriched in exosomes, which can be transferred to VSMCs, thus regulating the VSMC phenotype and participating in the entire AS process [74]. Zhang et al. reported that exosomal LINC01005 derived from ECs induced by ox-LDL promotes the VSMC phenotypic transition by regulating the miR-128-3p/KLF4 axis [75]. In addition, TET2 is a key factor in the VSMC phenotypic transition, as it protects ECs in AS, and exosomes in ECs transfer TET2 from ECs to VSMCs, slowing down the proliferation and migration of VSMCs and the formation of angiogenic intima. Inhibiting the EC-derived exosomal transfer of TET2 to VSMCs triggers platelet-derived growth factor (PDGF-BB)-induced phenotypic transition of VSMCs, promoting plaque formation and accelerating the development of AS [44].

Angiotensin-converting enzyme 2 (ACE2) is a promising cardiovascular target. Some research groups have reported that exosomes derived from endothelial progenitor cells downregulate the activated NF-kappaB pathway by delivering functional ACE2, thereby reducing the phenotypic transition of VSMCs induced by angiotensin II (Ang II) [76]. This finding indicates that ECs can communicate with VSMCs through exosomes at the precursor stage. Furthermore, vascular aging in diabetic patients is an important cause of AS. The vascular response under high glucose conditions is mainly related to signal transmission between ECs and VSMCs. Studies have shown that circRNA0077930 in exosomes secreted by the human vascular endothelium inhibits the expression of miR-622 and accelerates the aging of VSMCs [77]. In addition, data suggest that exosomal Notch3 derived from ECs regulates calcification and senescence in VSMCs through the mTOR signaling pathway under high glucose conditions [78].

At the same time, VSMC-derived exosomes can affect the function of ECs. VSMC-derived exosomes transduced by KLF5 are absorbed by ECs, enhancing the expression of miR-155 to help exosomes transfer miR-155 from VSMCs to ECs. However, overexpression of this VSMC-secreted exosomal miR-155 inhibits the proliferation and migration of ECs and reduces expression of the TJ glycoprotein, thus damaging endothelial barrier function as well as promoting the occurrence of AS [79].

Exosomes play an important role in information exchange between ECs and VSMCs. As molecular carriers, miR-143/145, miR-622, and miR-155 regulate AS by affecting message switching between ECs and VSMCs. Therefore, this provides a new direction for treating AS by studying the mechanism of inducing or inhibiting the communication between ECs and VSMCs mediated by exosomes.

## Roles of exosomes in the regulation of the interaction between macrophages and VSMCs

Many types of cells play different roles in the development of AS. However, macrophages and VSMCs are dominant in terms of numbers. During the phenotypic transition, VSMCs acquire macrophage characteristics and express macrophage markers, such as CD68 [80]. Both have high plasticity and can phagocytose ox-LDL into lipid-rich foam cells, thus aggravating AS [81]. It has also been demonstrated that foam cells and necrotic cores of plaques are derived from macrophages and VSMCs [82], indicating a close relationship between these two cell types. Therefore, the activity of macrophages and VSMCs is critical to the progression and outcome of AS, and understanding the interactions and molecular changes between them could be a boon for patients suffering from AS.

Macrophage-derived exosomes promote the VSMC phenotypic transition by activating the c-Jun/AP-1 signaling pathway [83] and triggering the expression of MMP-2 in VSMCs through the JNK and P38 pathways [84], both of which accelerate the AS process. New et al. proposed that pro-inflammatory macrophages release exosomes rich in phosphatidylserine membrane adhesion protein 5 and S100A9 to promote the transition of VSMCs into the osteoblast phenotype [85]. Moreover, macrophage-derived foam cells secrete more exosomes than macrophages to regulate the actin cytoskeleton and adhesion pathway, transport information molecules to VSMCs, and promote phosphorylation of the ERK and Akt pathways in VSMCs in a time-dependent manner, which promotes adhesion and migration of VSMCs [86].

To sum up, exosomes regulate the interactions between macrophages and VSMCs through multiple pathways. Therefore, regulating the status of VSMCs by targeting related exosomes to control the activities of macrophages and macrophage-derived foam cells can prevent AS. For example, exosomes derived from human mesenchymal stromal cells inhibit the activation of macrophages through miR-147 [87].

## Roles of exosomes in the regulation of the interactions between VSMCs

**Fig. 3:**
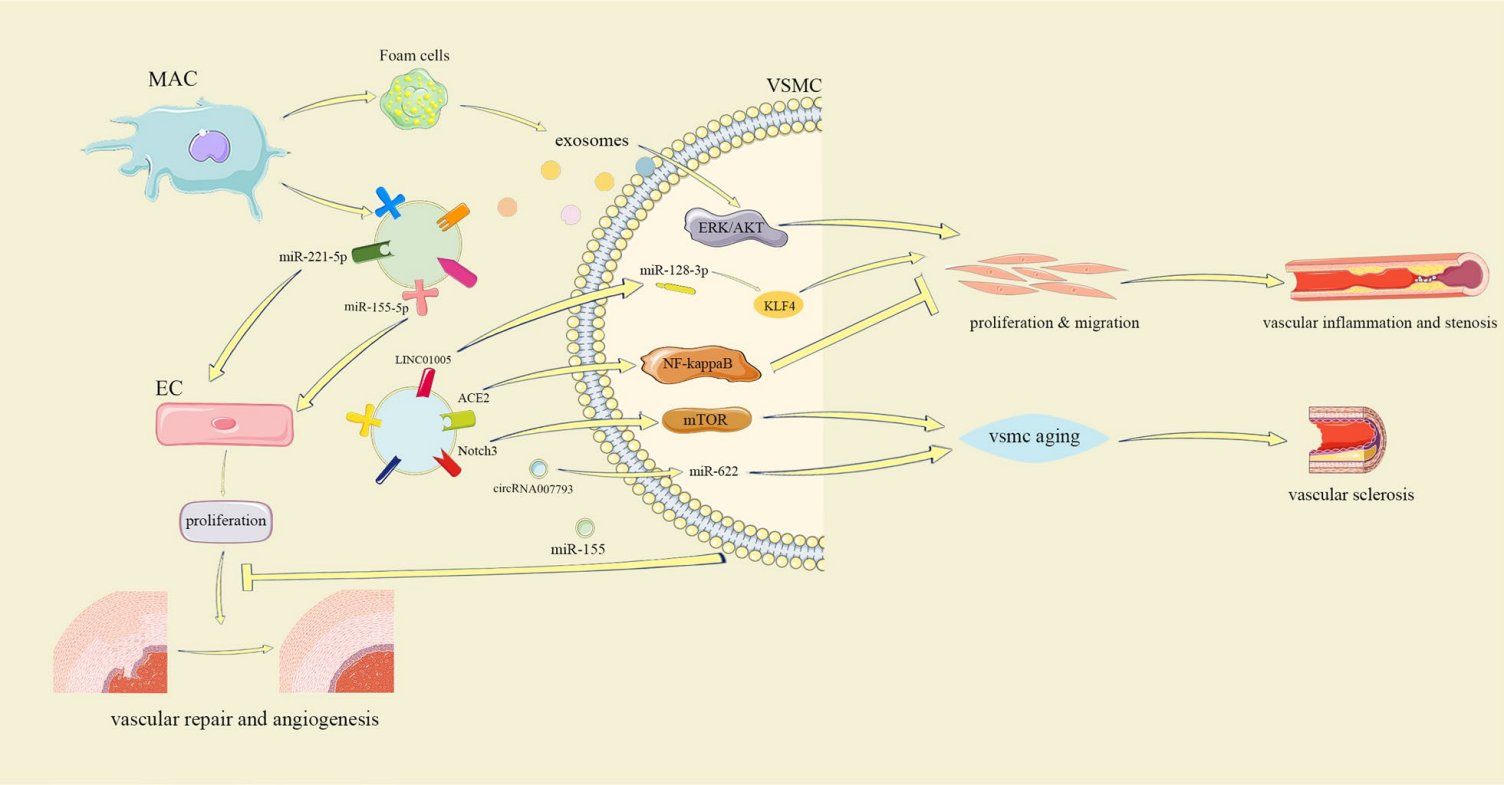
Macrophage-derived exosomal miR-155-5p and miR-221-5p promote EC proliferation to help vascular angiogenesis. EC-derived exosomal circRNA007793 inhibits miR-622 to promote cell aging. LINC01005 promotes proliferation and migration of VSMCs by regulating the miR-128-3p/KLF4 axis, while ACE2 down-regulates the activated NF-kappaB pathway to inhibit this process. Notch3 promotes the aging of VSMCs to ease vasosclerosis through the mTOR signaling pathway. VSMC-derived exosomal miR-155 inhibits EC proliferation to prevent damage to the vascular endothelium

Proliferation, migration, apoptosis, and calcification of VSMCs play an important role in AS, and their interactions are crucial for maintaining vascular wall balance. In vitro studies have shown that the effect of XBP1S on VSMCs controls the migration of ECs through exosomal miR-150 derived by VSMCs and the VEGFR/PI3K/Akt pathway driven by miR-150, thus regulating the maintenance of vascular homeostasis [88]. This finding indicates that the interactions between VSMCs and VSMCs may also involve intermediary cells. However, it is unclear whether VSMCs can directly regulate the phenotype of the same cell type through exosomes (Fig. 3).

## Discussion

VSMCs are highly specialized, highly plastic cells that exchange information with other cells and change into different phenotypes under the regulation of peripheral signaling molecules. The phenotypic transition ability of VSMCs is an inherent characteristic of this type of cell, which evolved in higher organisms. For example, during the early stage of AS, VSMCs switch to the proliferative migration phenotype, promoting intima neogenesis and vascular repair, while in the late stage of plaque, VSMCs switch to the osteoblast phenotype, promoting complete plaque calcification and increasing plaque stability. Therefore, this ability of VSMCs confers a survival advantage.

Exosomes may help identify novel therapeutic targets to promote vascular repair, enhance the stability of plaque, and promote the establishment of collateral circulation, which maximizes the beneficial effects of the VSMC phenotypic transition. Inhibiting the expression of exosomal miR-92a-3p, miR-21-3p, miR-663, and miR-222, and promoting the expression of exosomal miR-133, miR-143/145, and miR155-5p effectively restrain the proliferation and migration of VSMCs and improves vascular remodeling in AS. Regulation of exosomal LncRNA H19, miR-103-3p, miR-133a, miR-204/miR-211, and miR-34a may control the phenotypic transition from VSMC to osteoblast, effectively regulating vascular aging, calcification, and plaque stability in AS. Exosomal miR-146a is expected to play a positive role in protecting VSMCs from osteogenic differentiation and alleviating vascular calcification by targeting the increase of TXNIP in VSMCs [89] and simultaneously reducing the expression of osteogenic genes and reactive oxygen species. Exosomal miR-106a, miR-106a-3p, circHIPK3, miR-106a-5p, and miR-125b-5p are closely related to apoptosis of VSMCs. Intervening in their expression during different stages of AS may balance the proportion of apoptotic cells, maintain vascular elasticity, and reduce the degree of obstruction. New therapeutic targets to fundamentally control the phenotypic transition of VSMCs and the disease progression of AS are expected.

In addition to the studies described above, two recent studies have suggested the therapeutic potential of exosomes. Ke et al. suggested that endothelial colony-forming cell-derived exosomes regulate lipid homeostasis, activate autophagy, attenuate vascular endothelial injury, and play a protective role in AS [90]. Zhang et al. reported that mesenchymal stem cell-derived exosomes fight against damaged ECs induced by ox-LDL and restore vascular activity by fetal-lethal non-coding developmental regulatory RNA [91]. In addition, exosomes have a lipid bilayer membrane structure, which protects the encapsulated substances and targets specific cells or tissues. Therefore, exosomes are a well-targeted drug delivery system with bright prospects in precision medicine.

In addition to these therapeutic effects, exosomes are promising biomarkers for diagnosing and predicting AS. The measurement of exosomal miRNAs indicated [92] that exosomes of healthy individuals do not carry a significant number of miRNAs, which is to say that diseases may occur because a large number of exosomes with similar functions work together, so a significant increase in the number of particular miRNAs can be detected. Moreover, exosomes are easy to isolate, carry AS-specific signaling molecules, and are more sensitive and specific than miRNAs in the circulating blood [93]. Many studies have suggested that exosomes and their encapsulated miRNAs have diagnostic potential in AS. For example, miR-16-5p, miR-486-5p, and miR-30c-5p are associated with the recurrence of ischemic events after carotid atherosclerosis [94], while miR-30e and miR-92a are negatively correlated with plasma cholesterol levels and are upregulated in AS [95].

Exosomes have stimulated new ideas for preventing and treating AS, but also bring new challenges. For example, most studies on the communication between specific types of cells have focused on the one-way regulation of information transferred by exosomes from one cell type to another cell type. The interaction between cells is mutual, and the information exchange between two types of cells should also be bidirectional, particularly during AS in which ECs, VSMCs, and macrophages interact. They are upstream and downstream emissaries of each other and determine the progress and outcomes of AS together. Therefore, the study of exosomal communication between multiple cells has great prospects. Second, exosomes are involved in the metabolism, transport, and catabolism of lipids in vivo [96], and lipid accumulation and metabolism in VSMCs are also closely related to the development of AS; however, no study on exosomes in VSMC lipid metabolism is available. In addition, the balance point to regulate the phenotypic transition of VSMCs must be identified, and then the critical molecular mechanisms leading to ongoing AS will be found. However, how various types of cells respond to environmental molecules is poorly understood. When a specific exosomal component inhibitor is used, even if it has a target effect on VSMCs, it may have side effects on other surrounding cells, such as apoptosis, activation of various MMPs, or promoting the release of inflammatory mediators, which could lead to deterioration of end-stage AS and to serious cardiovascular events. Therefore, in addition to identifying the key exosomal components involved in this process, screening of specific pathways for the actions of these components on target cells is needed. More standardized in vitro isolation methods are needed to apply exosomes as diagnostic biomarkers.

## Conclusion

In brief, the pathogenesis of AS is not only dependent on a single change in a particular cell but is affected by a variety of pathological changes in multiple cell types. The phenotypic transition of VSMCs is an important process in the development of AS, as well as a major factor affecting AS vascular wall lesions. Several studies have confirmed that targeting the response of VSMCs by exosomes can prevent or aggravate AS. Therefore, VSMCs can be used as a gene therapy guide vector to target cells. The study of the phenotypic transition of VSMCs regulated by exosomes will provide a new direction for preventing, diagnosing, treating, and prognosing AS. Future studies should focus on validating specific exosomal components as biomarkers for detecting an early risk of AS and finding novel strategies for treating AS with exosomal components that target VSMCs. Furthermore, more research is needed to address other challenges posed by exosomes.

## Data Availability

Not applicable.

## Abbreviations

AS: Atherosclerosis
VSMC: Vascular smooth muscle cell
EC: Endothelial cell
MAC: Macrophage
MVB: Multivesicularbody
MSC: Mesenchymal stem cell
HUVEC: Human umbilical vein endothelial cell
PASMC: Pulmonary artery smooth muscle cell
ESC: Embryonic stem cell
EPC: Endothelial progenitor cell
LPS: Cytosolic lipopolysaccharide
α-SMA: α-Smooth muscle actin
SMMHC: Smooth muscle myosin heavy chain
ACE2: Angiotensin-converting enzyme 2
ECM: Extracellular matrix
TNBS: Trinitrobenzene sulfonic acid
HAVIC: Human aortic valve interstitial cell
EMT: Epithelial-mesenchymal transition
DCM: Dilated cardiomyopathy
HAEC: Human aortic endothelial cell
CMVEC: Cardiac microvascular endothelial cell
BMSC: Bone marrow mesenchymal stem cell
CMEC: Cardiac microvascular endothelial cell
Ang II: Angiotesin II
ox-LDL: Oxidized low-density lipoprotein
